# Anesthetic practices and physiological foundations in fetal surgery: a literature review

**DOI:** 10.31744/einstein_journal/2026RW1538

**Published:** 2026-02-12

**Authors:** Marco Augusto Sperandeo Liguori, Ana Carolina Florentino Lobato, Gustavo Yano Callado, Cid Akihiko Ura Kusano, Eduardo Félix Martins Santana

**Affiliations:** 1 Hospital Israelita Albert Einstein Faculdade Israelita de Ciências da Saúde Albert Einstein São Paulo SP Brazil Faculdade Israelita de Ciências da Saúde Albert Einstein, Hospital Israelita Albert Einstein, São Paulo, SP, Brazil.; 2 Hospital e Maternidade Santa Joana Department of Anesthesiology São Paulo SP Brazil Department of Anesthesiology, Hospital e Maternidade Santa Joana, São Paulo, SP, Brazil.; 3 Universidade Federal de São Paulo Department of Obstetrics São Paulo SP Brazil Department of Obstetrics, Universidade Federal de São Paulo, São Paulo, SP, Brazil.

**Keywords:** Anesthesia, Perinatology, Fetal therapies, Physiology

## Abstract

Fetal anesthesia plays a critical role in ensuring maternal and fetal safety and optimizing outcomes during fetal surgical interventions. Despite its importance, there is currently no consensus regarding optimal anesthetic strategies for these procedures. This narrative review explores the principal types of fetal surgery, including minimally invasive and fetoscopic procedures, open fetal surgery, and Ex Utero Intrapartum Treatment, with particular emphasis on the factors influencing anesthetic decision-making. A comprehensive literature search was conducted across SciELO, PubMed, and Google Scholar, including articles published in any language up to May 2024. This review aims to highlight current anesthetic techniques and relevant maternal-fetal physiological considerations. Although fetal surgery can be performed safely using different anesthetic modalities, anesthetic management should be individualized according to maternal and fetal conditions, patient preferences, and the expertise of the multidisciplinary team. The lack of standardized guidelines and the limited availability of high-quality evidence underscore the need for further research. Future studies should focus on protocol development, complication management, and the use of adjuvant therapies to enhance maternal and fetal outcomes.

## INTRODUCTION

Fetal medicine has advanced substantially over recent decades, offering new therapeutic options for complicated pregnancies and improved management of fetal malformations. Within this evolving field, fetal anesthesia plays a critical role in ensuring fetal safety, comfort, and physiological stability, while optimizing maternal-fetal outcomes. Despite its acknowledged importance, maternal-fetal monitoring and anesthetic management during fetal interventions remain heterogeneous, and no standardized protocols or universal consensus currently exist.

This review aims to examine the principal categories of fetal surgical interventions, minimally invasive and fetoscopic procedures, open fetal surgery, and Ex Utero Intrapartum Treatment (EXIT) procedures, and the anesthetic approaches that enable their safe performance.^(1,2)^ In addition, we review the relevant maternal and fetal physiological considerations associated with each surgical modality, highlighting their implications for anesthetic decision-making.

Finally, we describe the anesthetic techniques employed for each type of fetal intervention and discuss the key factors that must be considered during anesthetic planning, with particular emphasis on the unique characteristics of the maternal-fetal dyad. As fetal anesthesia represents a rapidly evolving and pioneering field in medicine, this review seeks to synthesize the most current evidence, delineate established practices, and identify gaps in knowledge that warrant further research to improve safety, quality of care, and perinatal outcomes.

## TYPES OF FETAL SURGERY

### Minimally invasive surgery

Minimally invasive fetal surgery is primarily performed using two techniques: fetoscopy and ultrasound-guided needle procedures. Ultrasound-guided procedures involve percutaneous insertion of a needle through the uterine wall, allowing targeted intervention under continuous ultrasound guidance.^(1)^ These procedures facilitate a wide range of therapeutic applications, with three primary indications: treatment of pleural effusions and congenital cystic adenomatoid malformations using thoracoamniotic shunts; relief of lower urinary tract obstruction via vesicoamniotic shunting; and selective feticide in complicated monochorionic diamniotic twin pregnancies, as practiced in many countries.^(2)^

Fetoscopic surgery may be performed using two approaches: a completely percutaneous technique or a laparotomy-assisted approach. In the percutaneous approach, trocars are inserted through the abdominal and uterine walls under ultrasound guidance. In the laparotomy-assisted approach, a large abdominal incision is performed, allowing externalization of the uterus and trocar placement under direct visualization.^(3)^ Fetoscopic techniques are classically employed for three main indications: laser photocoagulation of communicating placental vascular anastomoses in twin-to-twin transfusion syndrome; fetoscopic endoluminal tracheal occlusion in selected cases of congenital diaphragmatic hernia; and release of constricting bands in amniotic band syndrome.^(4,5)^ In addition, repair of myelomeningocele, traditionally performed via open hysterotomy, has increasingly been carried out using fetoscopic approaches over the past decade. Previous studies have demonstrated fetoscopic repair is associated with a reduction in maternal and fetal complications while preserving the therapeutic benefits observed with open surgical techniques.^(6)^ However, as highlighted in a recent systematic review, randomized controlled trials and robust long-term outcome data remain lacking, and no clear superiority has been established between open and fetoscopic repair techniques, reinforcing the need for individualized decision-making based on patient profile and institutional expertise.^(7)^

### Open surgery

Open fetal surgery is a highly invasive procedure that involves direct manipulation of the fetus through a transverse abdominal incision, providing exposure of the uterus. After uterine exposure, placenta mapping is performed and fetal positioning is optimized to facilitate access to the target anatomical structure, followed by creation of a hysterotomy. Throughout the procedure, amniotic fluid is continuously replaced with warmed crystalloid fluid using a high-flow infusion system.^(8)^ This approach has traditionally been employed for the treatment of two conditions: myelomeningocele and congenital pulmonary airway malformation.^(9)^

Open fetal surgery is generally indicated when minimally invasive and fetoscopic approaches are not feasible. When performed by experienced multidisciplinary teams, maternal and fetal outcomes may be comparable to less invasive methods. Nevertheless, open fetal surgery is associated with a higher risk of procedure-related complications, including premature rupture of membranes and preterm birth.^(10)^

### Ex utero intrapartum treatment

The EXIT procedure is indicated in cases of congenital malformations in which difficulty with fetal airway access is anticipated. This procedure allows the fetal airway to be secured, either by direct laryngoscopy or bronchoscopy with orotracheal intubation, or by tracheostomy, before fetoplacental separation occurs.^(11)^ By maintaining a prolonged period of uteroplacental circulation, the EXIT procedure minimizes the risk of fetal cardiopulmonary compromise. It allows potentially fatal neonatal emergencies to be managed in a controlled manner, thereby improving perinatal outcomes.

Indications for the EXIT procedure include congenital diaphragmatic hernia, intrathoracic masses, and severe cardiac malformations. Although effective, the EXIT procedure requires a highly skilled multidisciplinary team, and the potential risks of maternal morbidity must be carefully evaluated and balanced against anticipated fetal benefits.^(12)^

## PHYSIOLOGY

### Maternal physiological changes during fetal surgery

During fetal surgery, careful consideration of maternal physiological changes is essential to maintain hemodynamic stability and ensure safe anesthetic and perioperative management. By the second trimester of pregnancy, progesterone-mediated reduction in systemic vascular resistance results in an approximately 40% increase in cardiac output, a nearly 50% expansion in plasma volume, and an elevation in resting heart rate to approximately 100 beats per minute. Collectively, these adaptations have important implications for maternal blood pressure regulation.^(13–15)^ Notably, the expanded plasma volume may delay the clinical manifestation of hypovolemia, allowing parturients to tolerate blood loss of up to 1000 mL before overt signs develop.^(16)^

Pregnancy is also characterized by a hypercoagulable state, resulting from increased concentrations of clotting factors as well as mechanical factors related to uterine enlargement.^(17)^ Accordingly, appropriate thromboembolism risk stratification is essential, together with strict adherence to established perioperative and postoperative thromboprophylactic protocols.^(18)^

Maternal respiratory physiological changes also require careful attention. Increased maternal and fetal metabolic demands lead to rises in oxygen consumption and carbon dioxide production of up to 60% by late pregnancy. Progressive uterine enlargement displaces the diaphragm upwards, resulting in a reduction in functional residual capacity and predisposing to rapid oxygen desaturation following induction of general anesthesia.^(19–21)^ Accordingly, adequate pre-oxygenation, optimal patient positioning, and availability of multiple airway management devices are essential. Upper airway edema may further complicate endotracheal intubation, underscoring the importance of thorough preoperative airway assessment and preparation of alternative airway devices. Pregnant patients frequently demonstrate worsening Mallampati classification and an increased incidence of predictors associated with difficult airway management.^(22)^ In addition, increased intragastric pressure, decreased lower esophageal tone, and increased acid secretion contribute to an elevated risk of aspiration pneumonitis.^(23)^

Pregnancy-related metabolic changes result in a state of compensated respiratory alkalosis, which must be carefully maintained during mechanical ventilation to maintain optimal maternal and fetal perfusion.^(14,15,21)^ Furthermore, increased minute ventilation enhances the uptake of inhalational anesthetic agents, reducing the minimum alveolar concentration required to induce loss of consciousness by up to 30%.^(24)^

The fetus depends entirely on the uteroplacental interface for the exchange of oxygen, nutrients, and metabolic waste, rendering the preservation of maternal uterine blood flow critical. Because uterine blood flow lacks an intrinsic autoregulatory mechanism, it is directly dependent on maternal cardiac output and systemic blood pressure. Anesthetic interventions may reduce maternal blood pressure, necessitating frequent hemodynamic monitoring and readiness to administer vasopressors in the event of hypotension. In addition, maternal pain and uterine contractions induced by surgical manipulation can further compromise uterine blood flow, thereby requiring the use of tocolytic agents and uterine smooth muscle relaxants.^(25)^

Finally, the transplacental transfer of medications administered to the mother during fetal anesthesia must be carefully considered. Increased maternal sensitivity to anesthetic agents during pregnancy necessitates lower drug doses to achieve adequate anesthetic depths, whether administered via general anesthesia or neuraxial techniques.^(26)^ Administration of uterine-relaxing agents, including high concentrations of volatile inhalational anesthetics, magnesium sulfate, nitroglycerin, indomethacin, and terbutaline, may be required to suppress uterine contractions. However, the use of these agents must be carefully titrated to minimize maternal hypotension while preserving adequate uteroplacental perfusion and fetal oxygen delivery.^(27)^

### Fetal physiology and the concept of pain in its development

Pain is defined as a subjective sensory and emotional experience associated with actual or potential tissue damage.^(28)^ Historically, pain perception was believed to depend on conscious cortical interpretation of nociceptive stimuli transmitted through thalamocortical pathways, which are not fully functional before the end of the second trimester of gestation.^(29)^ However, emerging evidence suggests that fetuses may mount stress responses to noxious stimuli as early as 17 weeks of gestation.^(30–32)^

For pain perception to occur, several neurobiological prerequisites must be present. These include the presence and activation of peripheral nociceptors, which begin to develop by approximately 7 weeks of gestation, as well as the formation of the spinothalamic tract responsible for transmitting nociceptive and tactile information, and the development of the thalamus, which serves as the principal sensory relay center. In contrast, development of the cerebral cortex and its thalamocortical afferent connections begins later, between approximately 23 and 24 weeks of gestation.^(29,33)^ This delayed cortical development, together with the presence of endogenous neural inhibitory substances in fetal circulation, historically supported the view that fetuses were incapable of experiencing pain. More recent investigations, however, have demonstrated that prior to cortical maturation, functional neural connections extend to subcortical regions exhibiting organized topography closely resembling that of the cerebral cortex, between approximately 12 and 35 weeks of gestation.^(31)^ When considered alongside the development of peripheral nociceptive structures around the 17th week of gestation, these findings suggest that fetuses may possess the neurobiological capacity to experience pain at this developmental stage.^(30)^

Cortical development begins in the first trimester of pregnancy, as neurons migrate from the periventricular zone towards the cortical plate, initiating the formation of the cerebral cortex, including the insular cortex, a region implicated in pain perception in adults. Between approximately 24 and 32 weeks of gestation, thalamic afferent fibers progressively innervate the cortex, establishing thalamocortical connections that were historically considered essential, together with cortical maturation, for the conscious experience of pain.^(33)^

During the third trimester of gestation, an observational study of 13 fetuses demonstrated responses to potentially noxious stimuli, including facial expressions and limb movements.^(34)^ In addition, sympathetic activation accompanied by physiological and reflexive responses to such stimuli has been documented.^(33)^ Furthermore, fetal studies have shown reductions in stress-related hormones, including cortisol and adrenaline, following administration of fentanyl analgesia during invasive procedures.^(35)^

Some authors have argued that the presence of inhibitory neuroactive substances in fetal circulation may reduce the need for fetal analgesia, as these agents are thought to induce a degree of fetal sedation and a baseline sleep-like state. These substances, including adenosine, progesterone, allopregnanolone, and progesterone metabolites, act through multiple mechanisms to enhance GABAergic signaling, resulting in relative fetal immobility and analgesic effects.^(33)^ Nonetheless, evidence indicates that fetal behavioral state is not static, but instead cycles through varying levels of alertness that can shift towards more active states in response to external sensory stimulations. These observations, together with documented fetal responses to noxious stimuli, suggest that the fetus does not exist in a uniform state of perpetual sleep and retains the capacity for arousal and sensory responsiveness.^(36)^

Another important issue highlighted in the literature concerns the potential short- and long-term effects of anesthetic agents used during fetal surgery. In the acute setting, the primary concern is uteroplacental hypoperfusion resulting from maternal hypotension, most commonly related to vasodilation secondary to sympathetic blockade during neuraxial anesthesia.^(37,38)^ Anesthetic agents may also precipitate fetal bradycardia, either in conjunction with or independent of surgical manipulation and maternal blood loss, with the potential to progress to fetal hemodynamic collapse.^(39)^ Maternal and fetal hemodynamic alterations may be sufficient to induce fetal acidosis and subsequent myocardial depression, ultimately resulting in fetal distress.^(38)^ Therefore, preservation of fetal cardiovascular stability is a central consideration in anesthetic planning, and prompt recognition and management of these changes by the medical team are essential. Corrective measures may include maternal repositioning to prevent aortocaval compression, fluid administration, reduction of anesthetic depth, and use of vasopressor agents.^(40–42)^ In cases of sustained or refractory bradycardia, expedited delivery may be required, depending on fetal viability and the capabilities of the treating medical facility.^(1)^

In open fetal surgery, fetal resuscitation is most often achieved indirectly through maternal resuscitation, owing to the shared uteroplacental circulation. Interventions such as maternal volume expansion, repositioning to alleviate inferior vena cava compression, and administration of a vasopressor agent may restore fetal heart rate and improve uteroplacental perfusion.^(43)^ In rare but critical situations involving persistent fetal bradycardia or suspected fetal hemorrhage, intrauterine fetal transfusion may be indicated. This intervention consists of direct intravascular transfusion, most commonly via the umbilical vein, and requires specialized expertise in fetal intervention and continuous ultrasound guidance. In addition, direct fetal drug administration, including intramuscular atropine, vecuronium, and fentanyl, may be employed to support fetal cardiovascular stability and provide analgesia when significant fetal manipulation is anticipated or when ongoing fetal distress is detected.^(43)^

Long-term considerations must also address the potential risks of teratogenicity and neurotoxicity associated with anesthetic exposure, which may adversely affect postnatal neurodevelopment. Experimental studies have demonstrated anesthesia-induced neuroapoptosis in fetal rodent models^(44)^ and nonhuman primates.^(45,46)^ Findings in nonhuman primates are of particular relevance, given the closer similarity of their neurodevelopmental trajectory to that of humans.^(47)^ Moreover, these studies have shown a time-dependent pattern of neurodegeneration, with progressive changes observed following 2, 6, and 24 hours of anesthetic exposure.^(48)^ Despite these findings, clinically meaningful evidence in humans remains limited, and the mechanisms, magnitude, and long-term implications of potential anesthetic neurotoxicity continue to be actively investigated. To date, no conclusive human data have demonstrated definitive long-term teratogenic or neurodevelopmental harm attributable to fetal anesthetic exposure.^(49,50)^

Collectively, these maternal and fetal considerations underscore the complexity of fetal surgical interventions and highlight the necessity of a coordinated multidisciplinary approach. Successful management requires meticulous procedural planning, clearly defined intraoperative and postoperative contingency strategies, and appropriate institutional infrastructure capable of supporting the surgical procedure, maternal care, and, when necessary, immediate neonatal management.

## FETAL ANESTHESIA

Anesthetic planning for fetal surgery involves multiple considerations, most notably the extent of maternal and fetal tissue manipulation anticipated during the procedure. Fetal surgical interventions can be broadly classified into minimally invasive procedures, open fetal surgeries, and EXIT procedures (Table 1). Within the first two categories, careful attention must be paid to the specific surgical objectives, particularly whether the intervention involves direct fetal tissue manipulation. In addition, evaluation of maternal-fetal drug transfer is essential to determine whether fetal exposure achieved through maternal administration is sufficient or whether direct fetal drug administration is required as part of the anesthetic plan.^(51,52)^

**Table 1:** Anesthetic approaches, indications, and complications in fetal surgical interventions

Type of intervention	Approach	Indication - procedure	Fetal manipulation	Anesthesia	Complications
Minimally Invasive Surgery	Ultrasound-Guided	Reverse Twin Arterial Perfusion - Umbilical Cord Ablation by Radiofrequency	No	Local maternal anesthesia	Fetal injury, worsening of uterine contractions
	Fetoscopy	Twin-to-Twin Transfusion Syndrome - Laser Ablation of Placental Anastomoses	No	Sedation and neuraxial block	Maternal airway compromise
	Ultrasound-Guided	Congenital Cystic Adenomatoid Malformation - Thoracoamniotic Shunt	Yes	Sedation and neuraxial block with fetal anesthesia (intramuscular gluteal injection or intravenous via umbilical cord)	Maternal airway compromise
	Ultrasound-Guided	Lower Urinary Tract Obstruction - Vesicoamniotic Shunt	Yes	Sedation and neuraxial block with fetal anesthesia (intramuscular gluteal injection or intravenous via umbilical cord)	Maternal airway compromise
	Fetoscopy	Amniotic Band Syndrome - Amniotic Band Ligation	Yes	General anesthesia	Risks associated with maternal and fetal exposure to general anesthetics
	Fetoscopy	Diaphragmatic Hernia - Fetoscopic Endoluminal Tracheal Occlusion (FETO)	Yes	General anesthesia	Risks associated with maternal and fetal exposure to general anesthetics
	Fetoscopy	Myelomeningocele Repair	Yes	General anesthesia	Risks associated with maternal and fetal exposure to general anesthetics
Open Surgery	Hysterotomy	Myelomeningocele Repair	Yes	Sedation and neuraxial block for maternal anesthesia	Maternal airway compromise
	Fetoscopy	Myelomeningocele Repair	Yes	General anesthesia	Risks associated with maternal and fetal exposure to general anesthetics
EXIT	Hysterotomy	EXIT Procedure - Airway Management	Yes	General anesthesia	Risks associated with maternal and fetal exposure to general anesthetics

For optimal anesthetic planning, a thorough understanding of the placental transfer characteristics of each drug type is essential. Induction agents, particularly thiopental and propofol, readily cross the placenta due to their low ionization and lipid solubility.^(53)^ Inhalational halogenated agents are small, highly lipophilic molecules that reach the fetal circulation at low alveolar concentrations, necessitating cautious use due to potential cardiodepressive effects and concerns regarding fetal neurotoxicity.^(54)^ In contrast, neuromuscular blocking agents are generally large or highly ionizable molecules and exhibit minimal placenta transfer.^(1,53)^

Among opioids, morphine, fentanyl, and remifentanil are notable for their efficient placental passage and are commonly employed in fetal surgery.^(53)^ Remifentanil, in particular, is administered maternally to provide adequate maternal sedation while achieving sufficient fetal concentrations to promote analgesia and immobility, typically at low infusion rates of 0.1-0.2 *μ*g/kg/min.^(40,32)^

Additional anesthetic agents of interest include anticholinergics, primarily atropine; benzodiazepines; and sympathomimetics, all of which cross the placenta to varying degrees and should be considered based on procedural requirements.^(53)^

Fetal administration of analgesic and anesthetic agents is used to supplement maternal anesthesia during surgical procedures. This approach is indicated when maternal local or regional anesthesia is planned, but fetal tissue manipulation or fetal immobilization is required. Typically, fetal intramuscular injection of vecuronium or pancuronium at 0.2mg/kg, or rocuronium at 1mg/kg, is combined with fentanyl at 10-50*μ*g/kg, delivered either directly into the gluteal region or intravenously via the umbilical cord. Atropine at 20mcg/kg intramuscularly is often administered concurrently to counteract potential cardiodepressive effects. Alternatively, continuous maternal intravenous infusion of remifentanil can achieve comparable fetal analgesia and immobility. Fetal analgesia is generally unnecessary when the surgical target is the placenta or umbilical cord, as these organs lack nociceptors, provided fetal immobilization is adequately maintained.^(6,25,40,51)^

Intra-amniotic drug administration has been explored in animal models, demonstrating both safety and efficacy. However, data in humans are lacking, and appropriate dosing regimens have yet to be established.^(25)^

A 2022 systematic review encompassing 168 articles on fetal surgery compared complication rates associated with different anesthetic techniques. The review found no statistically significant differences between direct fetal drug administration and maternal administration via the placenta.^(55)^

Tocolytic agents are commonly employed during antenatal fetal interventions.^(56,57)^ The MOMS study provides a protocol for open fetal surgeries involving hysterotomy, which includes preoperative administration of 50mg of indomethacin orally or rectally, followed by repeated doses every 6 hours for 24 hours, then 25mg on the second day. Magnesium sulfate is initiated intraoperatively at a loading dose of 6 g, followed by maintenance at 2-4 g/hour for 18-48 hours postoperatively. Maintenance therapy with oral nifedipine, at 10-20mg every 4-6 hours, continues until term.^(57)^ In contrast, a 2018 systematic review suggested that indomethacin and magnesium sulfate alone may suffice for tocolysis in certain cases.^(58)^

For fetoscopic procedures, a more conservative pharmacological approach may be adopted, typically with preoperative prophylaxis using 100mg of indomethacin.^(59)^ Other agents, including atosiban and terbutaline, may also be employed, although terbutaline is associated with systemic side effects when initiated preoperatively.^(60)^ Inhalational anesthetics, such as sevoflurane, may be added when the patient undergoes general anesthesia.^(52,60)^ Despite these protocols, tocolytic protocols vary considerably, and no consensus currently exists; many of these medications carry potential adverse effects, some of which may be serious.^(58)^

### Minimally invasive procedures

In general, anesthesia for minimally invasive surgeries consists of light sedation combined with local anesthesia or neuraxial blockade, such as spinal anesthesia or epidural anesthesia. In selected cases requiring fetal intervention, including fetoscopic procedures for congenital malformations, maternal intravenous administration of remifentanil may provide adequate fetal analgesia and immobility, or a combination of agents may be administered directly to the fetus via intramuscular injection.^(25,40,51,52)^ General anesthesia may also be selected, as in certain cases of myelomeningocele repair, providing reliable fetal anesthesia but exposing the mother to the inherent risks of general anesthesia.^(6,61)^ When procedures involve direct fetal manipulation, intramuscular fetal administration of fentanyl, a neuromuscular blocker (e.g., rocuronium), and atropine should be considered to ensure analgesia and immobility, particularly when maternal general anesthesia is not used or is insufficient to achieve adequate fetal sedation.^(62)^

### Open surgery

Open fetal surgery is typically performed under general anesthesia, which may be combined with spinal or epidural anesthesia.^(52)^ Postoperative analgesia is particularly critical in this setting, as inadequate pain control may increase catecholamine release through autonomic activation, leading to uterine irritability and an elevated risk of preterm labor.^(57)^ To mitigate this risk, placement of an epidural catheter should be considered, as it provides effective blockade of nociceptive input, in contrast to the more limited modulation achieved with oral or intravenous analgesics and opioids alone.^(52)^

Invasive arterial blood pressure monitoring should be considered, given that many uterine relaxants are associated with peripheral vasodilation and hypotension. Accordingly, central venous access for the administration of vasopressors and intravenous fluids may be warranted to ensure adequate maternal hemodynamic stability and uteroplacental perfusion.^(63)^

Tocolysis represents another essential component of open fetal surgical management and is typically guided by institutional protocols. Adverse effects of tocolytic agents include hypotension, heart failure, and pulmonary edema. In light of the physiological pregnancy-related expansion of intravascular volume and the cardiovascular effects of tocolytics, fluid administration should be judicious, and urine output should be closely monitored to reduce the risk of pulmonary edema.^(58,64)^

## EXIT

Anesthetic planning for the EXIT procedure requires meticulous preoperative organization, with careful coordination of the distinct procedural stages and close involvement of a multidisciplinary team.^(65)^ Throughout the procedure, several critical phases demand specialized anesthetic management, necessitating close collaboration between the anesthesiologist and the multidisciplinary team.^(1)^

The selection of maternal anesthesia represents the initial and most consequential decision. EXIT procedures are most commonly performed under general anesthesia, using high concentrations of volatile agents to achieve uterine relaxation.^(66)^ Neuraxial anesthesia has been described as an alternative approach; however, in such cases, particular attention must be directed toward ensuring adequate fetal anesthesia and immobility.^(67)^ The anesthetic technique is selected primarily based on maternal considerations, including overall health status, medical history, anticipated airway difficulty, contraindications to regional anesthesia, and patient preferences.^(68)^

Tocolysis should be initiated immediately following the induction of maternal anesthesia using fast-acting agents with rapid systemic clearance.^(69)^ This is most commonly achieved with inhalational anesthetics such as desflurane and sevoflurane, either alone or in combination with adjunctive agents, most notably nitroglycerin.^(65)^ Alternative tocolytic agents include magnesium sulfate, calcium channel blockers^(69)^, indomethacin, and terbutaline.^(70)^ Nitroglycerin provides rapid and effective uterine relaxation but frequently necessitates vasopressor support to counteract maternal hypotension, which may compromise uteroplacental blood flow and fetal oxygen delivery.^(65,67)^ This risk is particularly relevant when maternal anesthesia is administered using neuraxial techniques.^(66)^

Following hysterotomy, the fetal head and neck are exteriorized, at which point continuous fetal monitoring should be initiated and a fetal anesthetic cocktail administered. Definitive fetal airway control must then be established using direct laryngoscopy, bronchoscopy, or surgical airway access, as indicated.^(69)^ Because fetal ventilation is not initiated until delivery is complete, maintenance of intact placental circulation via the umbilical cord is essential to preserve fetal oxygenation during this phase.^(1)^

After umbilical cord clamping, uterine relaxants and inhalational anesthetics, if used, should be promptly discontinued and replaced with alternative hypnotic agents, such as propofol, to maintain anesthesia. Concurrently, uterotonic agents, including oxytocin, methylergonovine, and misoprostol (prostaglandin E1), should be administered to promote uterine contraction and reduce the risk of postpartum hemorrhage.^(1,70)^

## SPECIFIC ANESTHETIC COMPLICATIONS IN FETAL SURGERY

Each anesthetic technique used in fetal surgery is associated with distinct maternal and fetal complications (Table 1). Maternal local anesthesia carries a risk of injury to the non-anesthetized and non-paralyzed fetus, provides no fetal analgesia, and does not induce uterine relaxation. Sedation combined with regional anesthesia may alleviate maternal anxiety and confer limited fetal analgesia; however, it increases the risk of aspiration due to the presence of an unprotected airway.^(25)^ Neuraxial regional anesthesia may be ineffective or result in a high or total spinal block and does not provide fetal analgesia, fetal anesthesia, or uterine relaxation.^(71)^ General anesthesia offers effective anesthesia for both the mother and fetus and facilitates uterine relaxation, but it is associated with technique-specific maternal and fetal risks. The combination of regional and general anesthesia, commonly employed in open procedures, incorporates the advantages and disadvantages of both techniques while improving postoperative analgesia.^(72)^

Administration of local anesthetics in a fetus with acidosis may result in ion trapping, leading to increased anesthetic concentrations in fetal circulation. In this context, ionized drug molecules become sequestrated within the fetal compartment, increasing the risk of adverse effects by limiting placental diffusion back to the maternal circulation. This phenomenon is most pronounced with cutaneous local anesthetics but is also relevant in the setting of neuraxial blockade.^(37)^

Pregnant patients are at increased risk of hypotension and hemodynamic instability following spinal anesthesia due to sympathetic blockade.^(73)^ Maternal hypotension also poses a significant risk to the fetus, as adequate placental perfusion is essential for fetal oxygenation. Consequently, careful monitoring and prompt management of maternal blood pressure are critical when neuraxial techniques are employed to prevent placental hypoperfusion and fetal compromise.^(74)^

Fetal cardiac output is highly dependent on heart rate, as the fetal myocardium contains a relatively limited amount of contractile tissue, rendering it particularly susceptible to the cardiodepressive effects of volatile anesthetic agents. These physiological characteristics predispose the fetus to hypotension and, in severe cases, cardiovascular collapse. Opioids may also induce fetal bradycardia; however, this effect is generally well tolerated provided maternal hemodynamic stability is maintained.^(40)^ When drugs are administered directly to the fetus via intramuscular injection, adjunctive medications are often included to attenuate these cardiovascular depressant effects. Nevertheless, resuscitation medications, including atropine (0.02mg/kg) and epinephrine (1*μ*g/kg), should be readily available prior to fetal drug administration.^(72)^

Propofol, another commonly used anesthetic agent, has also been associated with the development of fetal acidosis. When intravenous anesthesia is used as a sole technique, high cumulative doses of propofol may be required, and its context-sensitive half-life may result in prolonged fetal exposure. This pharmacokinetic profile may increase fetal risk if propofol is used in isolation. Accordingly, the use of multimodal anesthetic techniques is recommended to mitigate these effects, typically by combining low concentrations of volatile anesthetics (approximately 1%) with propofol to enhance hypnosis, reduce total propofol requirements, and contribute to uterine relaxation.^(75)^

When maternal hypotension occurs or is anticipated, pharmacologic support may be required to maintain adequate maternal arterial blood pressure and uteroplacental perfusion. Vasopressors commonly used in obstetric anesthesia, including ephedrine, metaraminol, norepinephrine, and phenylephrine, are considered effective and safe when administered appropriately.^(63,70)^

## CONCLUSION

Fetal surgical procedures can be performed safely using a range of anesthetic modalities when appropriately planned and executed. In collaboration with the patient, the anesthesiologist should select techniques that prioritize maternal and fetal safety, provide effective postoperative analgesia, and facilitate timely recovery for both mother and fetus. This decision-making process should incorporate patient preferences, maternal clinical status, gestational considerations, the anesthesiologist's expertise with specific techniques and pharmacologic agents, and the multidisciplinary team's capacity to manage perioperative complications.

As a comparatively emerging field, fetal surgery and its anesthetic management present substantial opportunities for further research aimed at improving safety, standardizing protocols, and refining anesthetic modalities and adjunctive therapies. This review highlights the importance of aligning anesthetic strategies with surgical approaches while identifying gaps in the literature that warrant further investigation. Although ethical and practical challenges limit experimental research in pregnant populations, continued advancement in this area has the potential to significantly enhance patient safety and clinical outcomes for both mothers and anesthesiologists involved in fetal surgery.

## Data Availability

The underlying content is contained within the manuscript.
